# Comparative genomics of *Coniophora olivacea* reveals different patterns of genome expansion in Boletales

**DOI:** 10.1186/s12864-017-4243-z

**Published:** 2017-11-16

**Authors:** Raúl Castanera, Gúmer Pérez, Leticia López-Varas, Joëlle Amselem, Kurt LaButti, Vasanth Singan, Anna Lipzen, Sajeet Haridas, Kerrie Barry, Igor V. Grigoriev, Antonio G. Pisabarro, Lucía Ramírez

**Affiliations:** 1 Genetics and Microbiology Research Group, Department of Agrarian Production, Public University of Navarre, 31006 Pamplona, Navarre Spain; 2 URGI, INRA, Université Paris-Saclay, 78026 Versailles, France; 3 U.S.Department of Energy Joint Genome Institute, Walnut Creek, CA 94598 USA

**Keywords:** Boletales, Brown-rot, Basidiomycete, Genome, Annotation, Transposable elements, Retrotransposon

## Abstract

**Background:**

*Coniophora olivacea* is a basidiomycete fungus belonging to the order Boletales that produces brown-rot decay on dead wood of conifers. The Boletales order comprises a diverse group of species including saprotrophs and ectomycorrhizal fungi that show important differences in genome size.

**Results:**

In this study we report the 39.07-megabase (Mb) draft genome assembly and annotation of *C. olivacea*. A total of 14,928 genes were annotated, including 470 putatively secreted proteins enriched in functions involved in lignocellulose degradation. Using similarity clustering and protein structure prediction we identified a new family of 10 putative lytic polysaccharide monooxygenase genes. This family is conserved in basidiomycota and lacks of previous functional annotation. Further analyses showed that *C. olivacea* has a low repetitive genome, with 2.91% of repeats and a restrained content of transposable elements (TEs). The annotation of TEs in four related Boletales yielded important differences in repeat content, ranging from 3.94 to 41.17% of the genome size. The distribution of insertion ages of LTR-retrotransposons showed that differential expansions of these repetitive elements have shaped the genome architecture of Boletales over the last 60 million years.

**Conclusions:**

*Coniophora olivacea* has a small, compact genome that shows macrosynteny with *Coniophora puteana*. The functional annotation revealed the enzymatic signature of a canonical brown-rot. The annotation and comparative genomics of transposable elements uncovered their particular contraction in the *Coniophora* genera, highlighting their role in the differential genome expansions found in Boletales species.

**Electronic supplementary material:**

The online version of this article (10.1186/s12864-017-4243-z) contains supplementary material, which is available to authorized users.

## Background

*Coniophora olivacea* is a basidiomycete fungus belonging to the order Boletales. *C. olivacea* produces brown-rot decay on dead wood of conifers (softwood) and, less frequently, on hardwood species. In addition, *C. olivacea* also damages wood buildings or construction materials. The genome sequence of its sister species *C. puteana* was made public in 2012 [1] and contributed to the understanding of genomic differences between brown and white-rot fungi. White-rot fungi are efficient lignin degraders, whereas brown-rot fungi attack cell wall carbohydrates leaving lignin undigested. The main responsible of this behavior are lignin-degrader peroxidases, which are abundant in white-rot species and particularly contracted in brown-rot and mycorrhizal fungi [2]. The Boletales order comprises a diverse group of species including saprotrophs and ectomycorrhizal species such as *Suillus sp.* or *Pisolithus sp.* During the last 6 years, up to 12 Boletales genomes have been sequenced and annotated [1, 3, 4]. Information that emerged from these studies showed important differences in genomic characteristics between the species belonging to this group, whose predicted common ancestor was dated 84 million years ago. Evolution from this boletales ancestor (supposed to be a brown-rot saprotroph) lead to the diversification and the appearance of ectomycorrhizae, which shows a particular contraction of the number of plant cell wall-degrading enzymes coding genes (PCWDE) [4, 5]. In addition, Boletales show important differences in their genome size and gene content. For example, the smallest assembled Boletales genome spans 38.2 Mb and has 13,270 annotated genes (*Hydnomerulius pinastri*), but the largest (*Pisolithus tinctorius*) spans 71.0 Mb and has 22,701 genes [4]. Previous studies in saprophytic basidiomycetes have shown that species with higher genome sizes tend to have more transposable elements [6]. Also, it has been described that species associated with plants (pathogenic and symbiotic) have genomes with expanded TE families [1, 7], although this trend varies between the three basidiomycete phyla [8]. In this paper, we describe the draft genome sequence and annotation of the brown-rot *C. olivacea*, and we compare it with the genomes of *C. puteana* as well as with that of three other Boletales showing important differences in genome sizes (*Serpula lacryman*s, *Pisolithus tinctorius* and *Hydnomerulius pinastri*). The results show that *C. olivacea* displays enzymatic machinery characteristic of brown-rot fungi encoded in a compact genome, carrying a small number of repetitive sequences. The comparative analysis with other Boletales shows that both ancient and modern LTR-retrotransposon amplification events have greatly contributed to the genome expansion along the evolution of Boletales.

## Methods

### Fungal strains and culture conditions

*Coniophora olivacea* MUCL 20566 was obtained from the Spanish Type Culture Collection and was cultured in SMY submerged fermentation (10 g of sucrose, 10 g of malt extract and 4 g of yeast extract per litre).

### Nucleic acid extraction

Mycelia were harvested, frozen, and ground in a sterile mortar in the presence of liquid nitrogen. High molecular weight DNA was extracted using the phenol-chloroform protocol described previously [9]. DNA sample concentrations were measured using a Qubit® 2.0 Fluorometer (Life Technologies, Madrid, Spain), and DNA purity was measured using a NanoDrop™ 2000 (Thermo-Scientific, Wilmington, DE, USA). DNA quality was verified by electrophoresis in 0.7% agarose gels. Total RNA was extracted from 200 mg of deep-frozen tissue using Fungal RNA E.Z.N.A Kit (Omega Bio-Tek, Norcross, GA, USA), and its integrity was verified using the Agilent 2100 Bioanalyzer system (Agilent Technologies, Santa Clara, CA, USA).

### Genome and transcriptome sequencing and assembly

A detailed description is provided in Additional file 1: Text S1. Briefly, the *C. olivacea* MUCL 20566 genome was sequenced using Illumina HiSeq-1 TB Regular 2 × 151 bp 0.309 kb. Sequenced reads were QC filtered for artifact contamination using BBDuk from the BBMap package (https://sourceforge.net/projects/bbmap/) and subsequently assembled with Velvet 1.2.07 [10]. The result -pair library with an insert size of 3000 +/− 300 bp *in silico* that was then assembled together with the original Illumina library with AllPathsLG [11]. Raw sequences were deposited in SRA (Sequence Read Archive) NCBI database under accession number SRP086489. Strand-specific RNASeq libraries were created and quantified by qPCR. Sequencing was performed using an Illumina HiSeq-2500 instrument. Reads were filtered and trimmed to remove artifacts and low quality regions using BBDuk. Transcriptome was de novo assembled using Trinity [12] and used to assist annotation and assess the completeness of the corresponding genome assembly using alignments of at least 90% identity and 85% coverage.

### Whole-genome alignment

The genome assemblies of *C. olivacea* MUCL 20566 and *C. puteana* (http://genome.jgi.doe.gov/Conpu1/Conpu1.home.html) were aligned using the Promer tool from the MUMmer 3.0 package [13]. Genome rearrangements were identified in the alignment with dnadiff tool from the same package.

### Genome annotation

The annotation of the *C. olivacea* MUCL 20566 assembly was performed using the Joint Genome Institute pipeline [14] to predict and functionally annotate protein-coding genes and other features such as tRNAs or putative microRNA precursors. The SECRETOOL pipeline [15] was used to identify putatively secreted proteins, considering the presence of signal peptides, cleavage sites, transmembrane domains and the GPI (glycosylphosphatidylinositol) membrane anchor. Carbohydrate-active enzymes (CAZys) were annotated based on BLAST [16] and HMMER [17] searches against sequence libraries and HMM (Hidden Markov Models) profiles of the CAZy database [18] functional modules. Protein structure predictions were carried out with Phyre2 [19]. Raw sequencing reads, genome assembly, transcriptome assembly, gene predictions and functional annotations are publicly available in the *C. olivacea* genome portal of Mycocosm database (http://genome.jgi.doe.gov/Conol1/Conol1.home.html).

### Annotation of transposable elements

Transposable elements (TEs) were identified and annotated in the *C. olivacea* assembly using REPET package [20, 21], as well as in the following boletales assemblies available in Mycocosm database (http://genome.jgi.doe.gov/programs/fungi/index.jsf): *Coniophora puteana* v1.0 (ID: Conpu1), *Hydnomerulius pinastri* v2.0 (ID: Hydpi2), *Serpula lacrymans* S7.3 v2.0 (ID: SerlaS7_3_2), *Pisolithus tinctorius* Marx 270 v1.0 (ID: Pisti1). Briefly, de novo TE detection was carried out with the TEdenovo pipeline [21] and the elements were classified with PASTEC [22]. The resulting TE library was fed into TEannot pipeline [20] in two consecutive iterations: the first one with the full library, and the second with an improved library consisting on consensus elements carrying at least one full-length copy after manually discarding false positives (i.e., *C. olivacea* genes).

### Insertion age of LTR-retrotransposons

Full-length LTR-retrotransposons were identified using LTRharvest [23] followed by BLASTX against Repbase [24]. Long Terminal Repeats were extracted and aligned with MUSCLE [25]. Alignments were trimmed using trimAl [26] and used to calculate Kimura’s 2P distances. The insertion age was calculated following the approach described in [27] using the fungal substitution rate of 1.05 × 10^−9^ nucleotides per site per year [6, 28].

### Identification of gene families

All-by-all BLASTP followed by MCL (Markov Cluster Algorithm) clustering [29] was carried out with *C. olivacea* protein models using a threshold value of e^−5^ and an inflation value of 2. We considered gene families carrying four or more genes for further analyses.

### Phylogenetic analyses

The predicted proteomes of the following species were downloaded from Mycocosm database (Mycocosm ID in parenthesis):

*Agaricus bisporus* var. *bisporus* H97 v2.0 (Agabi_varbisH97_2), *Boletus edulis* v1.0 (Boled1), *Coniophora olivacea* MUCL 20566 v1.0 (Conol1), *Coniophora puteana* v1.0 (Conpu1), *Cryptococcus neoformans* var. *grubii* H99 (Cryne_H99_1), *Fomitopsis pinicola* FP-58527 SS1 v3.0 (Fompi3), *Gyrodon lividus* BX v1.0 (Gyrli1), *Hydnomerulius pinastri* v2.0 (Hydpi2), *Leucogyrophana mollusca* KUC20120723A-06 v1.0 (Leumo1), *Paxillus involutus* ATCC 200175 v1.0 (Paxin1), *Phanerochaete chrysosporium* RP-78 v2.2 (Phchr2), *Pisolithus tinctorius* Marx 270 v1.0 (Pisti1), *Pleurotus ostreatus* PC15 v2.0 (PleosPC15_2), *Rhizopogon vinicolor* AM-OR11–026 v1.0 (Rhivi1), *Scleroderma citrinum* Foug A v1.0 (Sclci1), *Serpula lacrymans* S7.3 v2.0 (SerlaS7_3_2), *Suillus luteus* UH-Slu-Lm8-n1 v2.0 (Suilu3), *Trametes versicolor* v1.0 (Trave1). Species phylogeny was constructed as follows: all-by-all BLASTP followed by MCL clustering was carried out with a dataset containing the proteomes of all the species. The clusters carrying only one protein per species were identified, and the proteins were aligned using MAFFT [30]. The alignments were concatenated after discarding poorly aligned positions with Gblocks [31]. The phylogeny was constructed using RaxML [32] with 100 rapid bootstraps under PROTGAMMAWAGF substitution model. Phylogenetic reconstruction of Gypsy reverse-transcriptases was carried out as follows: Reverse transcriptase RV1 domains were extracted from LTR-retrotransposons of the TE consensus library using Exonerate [33] and aligned with MUSCLE. The alignments were trimmed using trimAl with the default parameters, and an approximate maximum likelihood tree was constructed using FastTree [34].

## Results

### *C. olivacea* assembly and annotation

The nuclear genome of *C. olivacea* was sequenced with 137 X coverage and assembled into 863 scaffolds accounting for 39.07 Mb, 90.3% of the genome size estimation based on k-mer spectrum (43.28 Mb). The mitochondrial genome was assembled into two contigs accounting for 78.54 kb. The assembly completeness was 99.78% according to the Core Eukaryotic Genes Mapping Approach (CEGMA [35]), with only one missing accession (KOG1322, GDP-mannose pyrophosphorylase). We assembled 66,567 transcripts (mean lenght = 2,744 nt, median = 2,154 nt) of which 97.8% could be mapped to the genome. The *C. olivacea* assembled genome was more fragmented than its close relative *C. puteana* (Table 1). The total repeat content was 2.91% of which 2.15% corresponded to transposable elements, 0.64% to simple repeats, and 0.12% to low complexity regions. The estimation of repeat content from low-coverage Illumina data (3.8X) yielded 6% of the genome size covered by transposable elements (Additional file 2: Table S1). We used transcriptomic information, *ab initio* predictions and similarity searches to predict a total of 14,928 genes—84.5% of them having a strong transcriptome support (spanning more than 75% of the gene length). In addition, 88.3% of the annotated genes had significant similarity to proteins from the NCBI nr database and 46.6% to the manually curated proteins from the Swiss-Prot database (cutoff e^−05^) [36]. A total of 7,841 predicted proteins (52.3%) carried Pfam domains and 1,471 (9.8%) carried signal peptide, of which 470 were predicted to be secreted using the more stringent SECRETOOL pipeline.

**Table 1:** Summary of *C. olivacea* genome assembly and annotation

Feature	*C. olivacea*	*C. puteana*
Genome assembly size (Mb)	39.07	42.97
Sequencing coverage depth	137.7×	49.5×
Number of scaffolds	863	210
Scaffold N50 ^a^	80	7
Scaffold L50 (Mb) ^b^	0.14	2.40
N° scaffold gaps	127	412
Genome assembly gaps (%)	0.24	2.57
Assembly completeness (%)	99.78	Unknown
Repeat content (%) ^c^	2.91	4.68
GC content (%)	52.82	52.4
Number of genes	14,928	13,761
Gene density (genes/Mb)	382.07	320.26
Predicted secreted proteins	470 (3.1%)	504 (3.7%)

^a^ N50 indicates the number of scaffolds that account for 50% of the total assembled sequence

^b^ L50 indicates that 50% of the total sequence is assembled in scaffolds larger than this size

^c^ Includes TE, simple repeats and low complexity regions

**Fig. 1:**
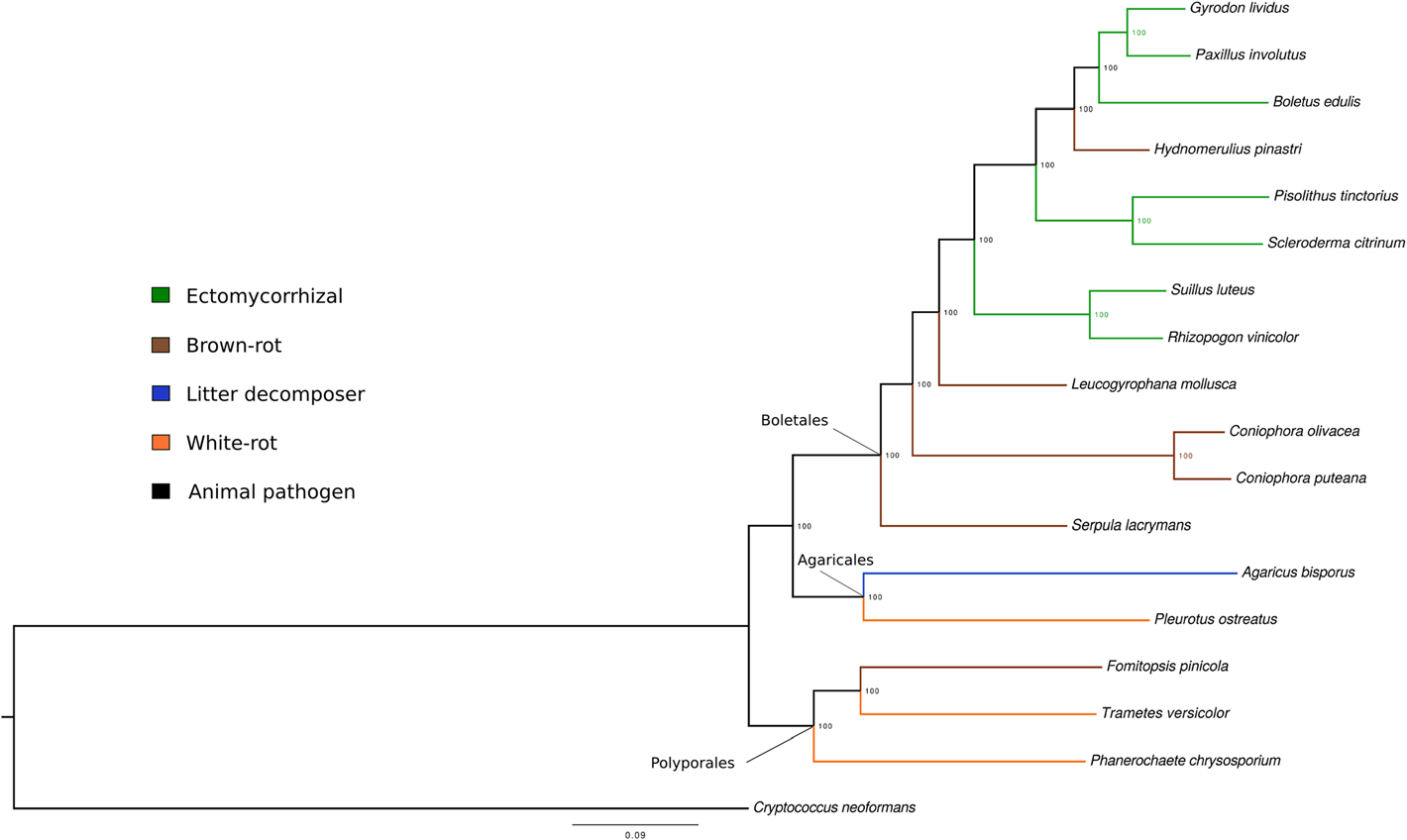
Maximum-likelihood phylogeny of 17 agaricomycetes inferred from 1677 genes. Branch labels indicate the results of 100 bootstraps

The multigene phylogeny based on 1,677 conserved single copy genes displayed different classes, orders and families in branches congruent with previous phylogenetic data [37] and with very high support. *C. olivacea* was placed in a branch next to its sequenced closer species *C. puteana* representing the Coniophoraceae family in the order Boletales (Fig. 1).

**Fig. 2:**
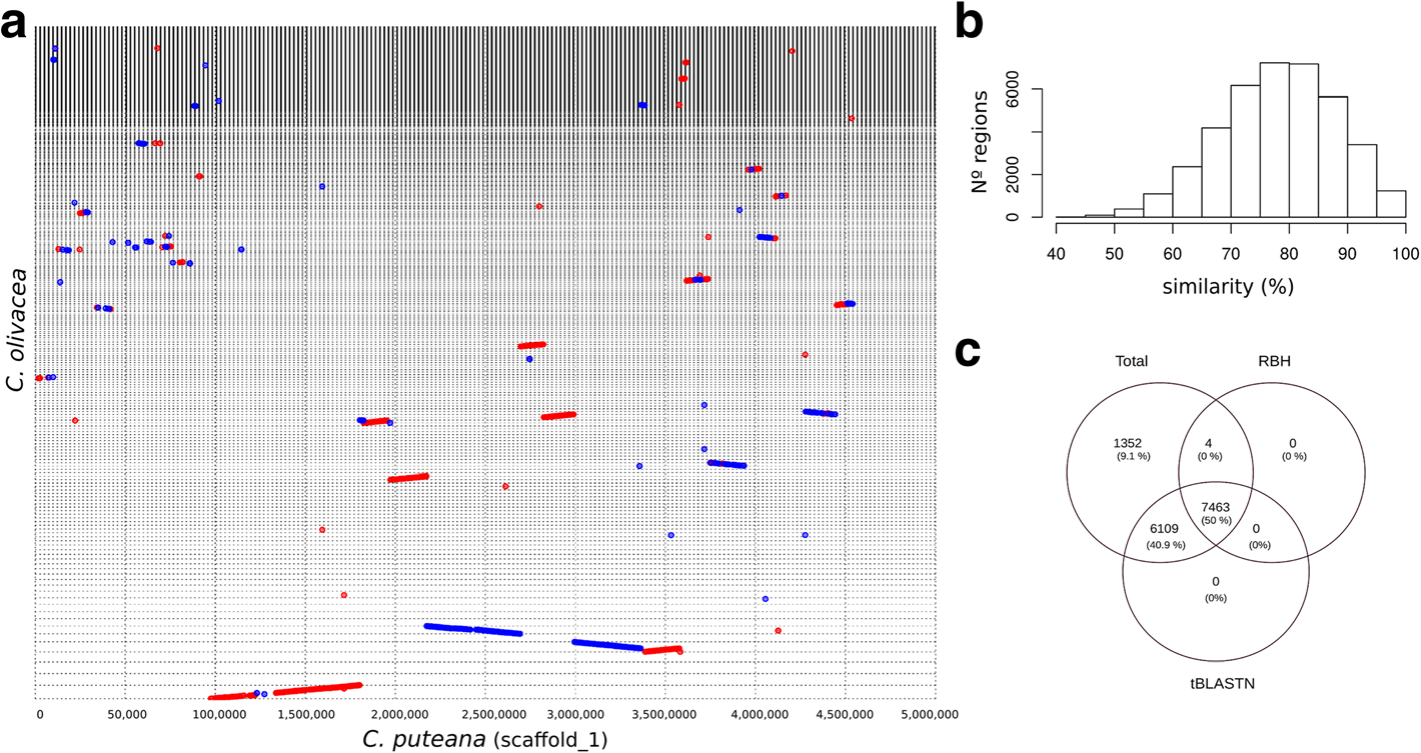
**a** Synteny dot plot showing a fraction of the whole-genome alignment between *C. puteana* and *C. olivacea*. Every grid line in the y-axes represents the end of one scaffold and the beginning of the next. Forward matches are displayed in red, while reverse matches are displayed in blue. **b** Histogram of similarity of the 39,506 aligned regions. **c** Venn diagram summarizing the amount of genes shared by the two genomes based on reciprocal best hit (RBH) and tBLASTN is shown in panel C

The whole-genome protein-based alignment between the two Coniophoraceae species spanned 52.7% of the *C. olivacea* and 48.0% of *C. puteana* assemblies. It shows evidence of macrosynteny between the two species (Fig. 2a, Additional file 3: Fig. S1), with an average similarity of 78.4% in the aligned regions (Fig. 2b) and numerous inversions (1,027 regions). The good conservation between both genomes in protein coding regions was evidenced by the amount of orthologous genes obtained using the reciprocal best hit approach (7,468 genes with more than 70% identity over 50% of protein sequences) and by the number of *C. olivacea* proteins yielding significant tBLASTN hits against the *C. puteana* genome (13,572 genes, cutoff e-5, Fig. 2c). For the remaining 1,352 *C. olivacea-*specific (orphan) genes, only 48 could be functionally annotated based on KOG (Eukaryotic Orthologous Groups), KEGG (Kyoto Encyclopedia of Genes and Genomes), GO (Gene Ontology) or InterPro databases.

### Carbohydrate-active enzymes of *C. olivacea*

The annotated proteome was screened for the presence of carbohydrate-active enzymes (CAZy). A total of 397 proteins were annotated and classified into different CAZy classes and associated modules. The CAZyme profile of *C. olivacea* was very similar to that of *C. puteana* although small differences were found in the glycoside hydrolases (GH, Additional file 4: Table S2). Some families such as GH5, GH18 or GH31 were smaller than in *C. puteana*. Similar to other brown-rot basidiomycetes, *C. olivacea* lacked Class II peroxidases (Auxiliar Activities AA2) and displayed a reduced set of other cellulolytic enzymes such GH6 (1), GH7 (1) and CBM1 (2) and AA9 (6).

### Functional characteristics of *C. olivacea* predicted secretome

Using SECRETOOL pipeline we predicted 470 putatively secreted proteins in *C. olivacea* and 504 in *C. puteana*. An enrichment analysis of gene ontology (GO) terms was performed to determine what gene functions were over-represented in the secreted proteins. Thirty GO terms were significantly enriched including 24 corresponding to molecular functions, four to biological processes and two to cellular components (Table 2). The most enriched molecular function was “feruloyl esterase activity,” which is responsible for plant cell-wall degradation. “Polysaccharide catabolic process” was the most enriched GO term within the biological processes, and “extracellular region” within the cellular components (Table 2).

**Table 2:** GO terms significantly enriched in the predicted secretome of *C. olivacea*

Molecular Function	Description	GO/Secretome	GO/Genome	*p* value^a^
GO:0030600	Feruloyl esterase activity	6/470	9/14,928	0.000171
GO:0042500	Aspartic endopeptidase activity, intra membrane cleaving	11/470	20/14,928	0.000192
GO:0008843	Endochitinase activity	8/470	14/14,928	0.000194
GO:0004568	Chitinase activity	8/470	14/14,928	0.000194
GO:0004650	Polygalacturonase activity	11/470	15/14,928	0.000354
GO:0004806	Triglyceridelipase activity	11/470	29/14,928	0.000376
GO:0016160	Amylase activity	25/470	40/14,928	0.000737
GO:0008933	Lytic transglycosylase activity	25/470	40/14,928	0.000737
GO:0015927	Trehalase activity	25/470	40/14,928	0.000737
GO:0015925	Galactosidase activity	25/470	40/14,928	0.000737
GO:0015924	Mannosyl-oligosaccharide mannosidase activity	25/470	40/14,928	0.000737
GO:0015929	Hexosaminidase activity	25/470	40/14,928	0.000737
GO:0015928	Fucosidase activity	25/470	40/14,928	0.000737
GO:0008810	Cellulase activity	9/470	11/14,928	0.00089
GO:0015926	Glucosidase activity	25/470	41/14,928	0.000948
GO:0015923	Mannosidase activity	25/470	41/14,928	0.000948
GO:0004620	Phospholipase activity	9/470	32/14,928	0.000968
GO:0004553	Hydrolase activity hydrolyzing O-glycosyl compounds	44/470	99/14,928	0.00105
GO:0004194	Obsolete pepsin A activity	17/470	42/14,928	0.00121
GO:0005199	Structural constituent of cell wall	16/470	33/14,928	0.00129
GO:0030246	Carbohydrate binding	9/470	25/14,928	0.00143
GO:0004190	Aspartic-type endopeptidaseactivity	20/470	44/14,928	0.00193
GO:0004099	Chitin deacetylase activity	5/470	9/14,928	0.00803
GO:0004185	Serine-type carboxypeptidase activity	5/470	12/14,928	0.0467
Biological Process				
GO:0000272	Polysaccharide catabolic process	5/470	6/14,928	0.000414
GO:0006508	Proteolysis	43/470	189/14,928	0.00128
GO:0005975	Carbohydrate metabolic process	65/470	161/14,928	0.00176
GO:0006629	Lipid metabolic process	10/470	50/14,928	0.00674
Cellular Component				
GO:0005576	Extracellular region	7/470	15/14,928	0.000354
GO:0005618	Cell wall	18/470	35/14,928	0.00224

^a^ Bonferroni corrected, Fisher *p*-value

### Analysis of putatively secreted multigene families

Using all-by-all BLASTP followed by MCL we clustered by similarity the 1,471 proteins carrying signal peptides in *C. olivacea*. We used all proteins carrying signal peptides rather than only SECRETOOL predictions in order to obtain larger protein clusters. Up to 60% of the 1,471 proteins grouped in clusters were formed by 2 to 59 genes (Additional file 5: Table S3), showing the same distribution as the whole proteome (*p* = 0.6032, Wilcoxon test, 61% of the 14,928 predicted genes were found in clusters containing 2 to 157 members). For further analysis of the secreted genes found in clusters, we focused on the 70 clusters (families) formed by four or more gene members. Using the KOG, KEGG, InterPro and GO databases, we could assign functions to 45 out of the 70 gene families (Table 3). Cytochrome P450, hydrophobins and aspartic-peptidases were the largest gene families. In addition, 17 CAZys clusters were found including glycoside hydrolases (GH), carbohydrate esterases (CE), carbohydrate-binding modules (CBMs) and redox enzymes classified as auxiliary activities (AA). 25 clusters lacked functional annotation, and some of them had a high number of genes (clusters 2, 6 and 7 in Table 3). All of these genes belonging to families with unknown function were further analyzed with Phyre2 to predict their protein structure and used for PSI-BLAST (Position-Specific Iterated BLAST) analysis. Using this approach, two gene families were functionally annotated with high confidence (96.3–97.4% confidence for individual protein predictions): one as a copper-dependent lytic polysaccharide monooxygenase (LPMO, also known as AA9; cluster 16), and the other as thaumatin-lyke xylanase inhibitor (*tlxi*, cluster 48). The Cluster16 containing putative LPMOs was particularly interesting. This was formed by 10 genes coding for small proteins ranging from 130 to 162 amino acids with three exons (with the exception of protein ID839457 that shows only two). All these genes coded for proteins that have a signal peptide but lack of known conserved functional domains. Six were confidently annotated as LPMOs by Phyre2, and four of them were predicted to be secreted by SECRETOOL. In addition, this family of unknown proteins is conserved in all the agaricomycetes shown in Fig. 1. Interestingly, four members of this family appear as a tandem located in *C. olivacea* scaffold_124 (scaffold_426:4800–12,000).

**Table 3:** Size and functional annotation of *C. olivacea* predicted gene families targeted to the secretory pathway

Gene family	SignalP	SECRETOOL	Functional annotation
Cluster_1	59	3	Cytochrome P450
Cluster_2	33	0	Unknown
Cluster_3	32	17	Hydrophobin
Cluster_4	19	11	Aspartic peptidase
Cluster_5	18	12	Carboxylesterase
Cluster_6	17	0	Unknown
Cluster_7	15	0	Unknown
Cluster_8	14	12	Peptidase G1
Cluster_9	14	9	RlpA-likelipoprotein
Cluster_10	13	0	Pheromone mating factor, STE3
Cluster_11	13	0	Unknown
Cluster_12	12	3	Peptidase S8/S53
Cluster_13	11	9	Unknown
Cluster_14	10	0	CAZy:GH18
Cluster_15	10	9	Cytochrome P450
Cluster_16	10	6	Unknown/lytic polysaccharide monooxygenase (LPMO/ CAZy:AA9)
Cluster_17	9	5	Asparticpeptidase
Cluster_18	9	5	CAZy:CE4 Carbohydrate Esterase Family 4
Cluster_19	9	0	CAZy:GH16
Cluster_20	9	2	Peptidase S10
Cluster_21	9	5	Sugar transporter
Cluster_22	9	4	Unknown/putative lipoprotein
Cluster_23	8	6	Fungal lipase
Cluster_24	8	0	Isoprenylcysteinecarboxylmethyltransferase
Cluster_25	8	0	Monooxygenase, FAD-binding
Cluster_26	7	7	Ser-Thr-rich glycosyl-phosphatidyl-inositol-anchored membrane family
Cluster_27	7	0	Unknown
Cluster_28	7	1	Unknown
Cluster_29	6	5	CAZy:GH128
Cluster_30	6	0	CAZy:GH28
Cluster_31	6	3	CAZy:GH3
Cluster_32	6	2	Peptidase M28
Cluster_33	6	6	Thaumatin
Cluster_34	6	2	Unknown
Cluster_35	6	6	Unknown
Cluster_36	5	1	Aspartic peptidase
Cluster_37	5	2	CAZy:AA1_1
Cluster_38	5	4	CAZy:AA5_1
Cluster_39	5	5	CAZy:AA9
Cluster_40	5	1	CAZy:CBM5
Cluster_41	5	4	CAZy:GH12
Cluster_42	5	5	CAZy:GH30_3
Cluster_43	5	0	CAZy:GH47
Cluster_44	5	2	CAZy:GH71
Cluster_45	5	0	Monooxygenase
Cluster_46	5	2	Unknown
Cluster_47	5	0	Unknown
Cluster_48	5	5	Unknown/xylanase inhibitor tl-xi
Cluster_49	5	4	Unknown
Cluster_50	5	4	Unknown
Cluster_51	5	4	Unknown
Cluster_52	5	0	Unknown
Cluster_53	5	4	Unknown
Cluster_54	5	0	Unknown
Cluster_55	5	0	Unknown
Cluster_56	4	0	CAZy:GH18, CAZy:CBM5
Cluster_57	4	0	CAZy:GH31
Cluster_58	4	3	CAZy:GH55
Cluster_59	4	4	Flavin monooxygenase-like
Cluster_60	4	3	GOLD
Cluster_61	4	2	Histidine phosphatase superfamily, clade-2
Cluster_62	4	3	Lysophospholipase
Cluster_63	4	1	Peptidase S28
Cluster_64	4	0	Proteolipid membrane potential modulator
Cluster_65	4	3	RlpA-like, ceratoplatanin
Cluster_66	4	1	Thioredoxin-like fold
Cluster_67	4	3	Unknown
Cluster_68	4	3	Unknown
Cluster_69	4	3	Unknown
Cluster_70	4	0	Unknown

Protein IDs of each cluster are shown in Additional file 5: Table S3

### Impact of repeat content on *C. olivacea* genome size and other Boletales

**Fig. 3:**
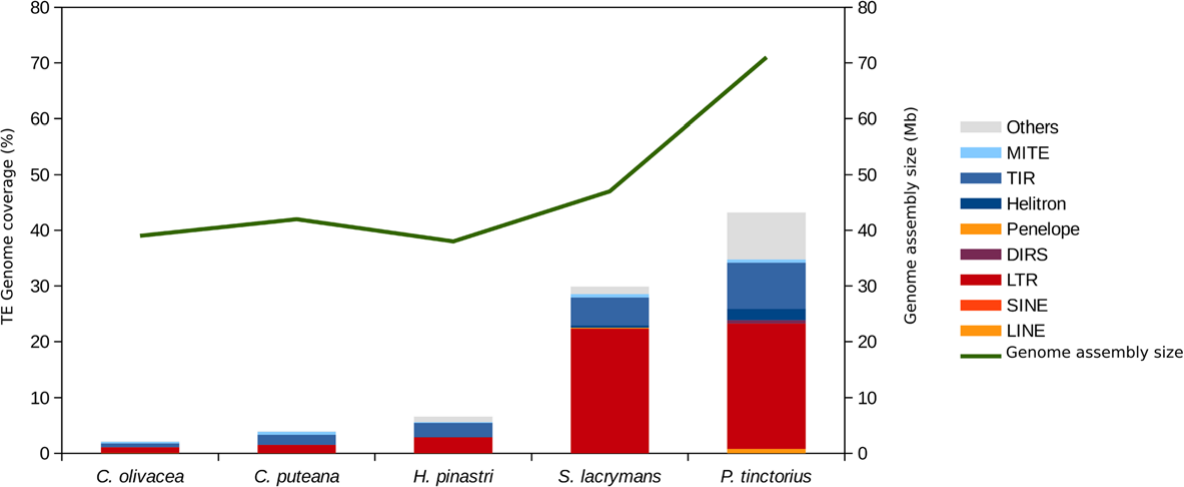
TE content and genome size in five Boletales species. TE content is shown as a histogram, and genome size as a green line in panel A. Panel B shows a histogram representing the number of TE families found in each species

To study the role that TEs have played in the evolution of the Boletales genomes, we annotated and quantified the TE content in five species showing important differences in genome size: *C. olivacea* (39.1 Mb), *C. puteana* (42.9 Mb) [1]*, Hydnomerulius pinastri* (38.2 Mb) [4], *Serpula lacrymans* (47.0 Mb) [3] and *Pisolithus tinctorius* (71.0 Mb) [4] (Additional file 6: Dataset S1, Additional file 7: Dataset S2, Additional file 8: Dataset S3, Additional file 9: Dataset S4, Additional file 10: Dataset S5). TEs were de novo identified and annotated using pipelines of the REPET package. The results yielded major differences in TE content between the five species, with *C. olivacea, C. puteana* and *H. pinastri* having low TE content (2.15%, 3.94% and 6.54% of their corresponding genome sizes), and *S. lacrymans* and *P. tinctorius* having up to 29.45% and 41.17% of their genomes occupied by TEs, respectively (Fig. 3, Table 4). In addition to higher TE content, species with larger genome assembly size showed higher TE diversity as reflected by the higher number of TE families, which ranged between 43 in *C. olivacea* to 432 in *P. tinctorius*.

**Table 4:** Summary of TE content in four Boletales genome assemblies

*Classification*	*C. olivacea*			*C. puteana*			*H. pinastri*			*S. lacrymans*			*P. tinctorius*		
	(43) families			(108) families			(87) families			(230) families			(432) families		
	Copies	Full copies	Coverage (%)	Copies	Full copies	Coverage (%)	Copies	Full copies	Coverage (%)	Copies	Full copies	Coverage (%)	Copies	Full copies	Coverage (%)
Class I															
LINE	30	4	0.03	0	0	0.00	0	0	0.00	0	0	0.00	317	41	0.80
LINE (unknown)	29	5	0.02	11	3	0.01	23	3	0.02	0	0	0.00	14	1	0.01
SINE	0	0	0.00	6	2	0.00	0	0	0.00	0	0	0.00	9	1	0.00
LTR/Copia	36	7	0.09	441	27	0.83	267	10	0.72	3,773	86	6.04	1,617	101	2.43
LTR/Gypsy	394	13	0.93	299	28	0.54	767	42	2.13	6,949	268	16.27	8,434	575	19.28
LTR/LARD	0	0	0.00	60	8	0.08	0	0	0.00	0	0	0.00	361	2	0.53
LTR/TRIM	15	4	0.02	136	4	0.08	0	0	0.00	0	0	0.00	576	93	0.20
DIRS	0	0	0.00	0	0	0.00	0	0	0.00	0	0	0.00	361	36	0.58
Penelope	0	0	0.00	0	0	0.00	0	0	0.00	69	11	0.15	0	0	0.00
Class II															
Helitron	0	0	0.00	0	0	0.00	21	4	0.04	260	25	0.43	1,386	38	2.01
TIR/DDE	361	28	0.52	362	38	0.68	1,089	55	2.16	2,148	166	3.04	3,366	255	4.25
TIR (unknown)	143	34	0.18	720	115	1.10	323	49	0.36	736	67	1.55	1,115	40	1.85
MITE	410	85	0.30	702	264	0.56	121	43	0.12	539	98	0.62	1,102	227	0.59
Maverick (putative)	0	0	0.00	0	0	0.00	0	0	0.00	0	0	0.00	56	3	0.21
Unknown	67	4	0.07	99	9	0.06	865	133	0.99	1,138	167	1.34	8,611	708	8.44
TOTAL	1,485	184	2.15	2,836	498	3.94	3,476	339	6.54	15,612	888	29.45	27,325	2,121	41.17

**Fig. 4:**
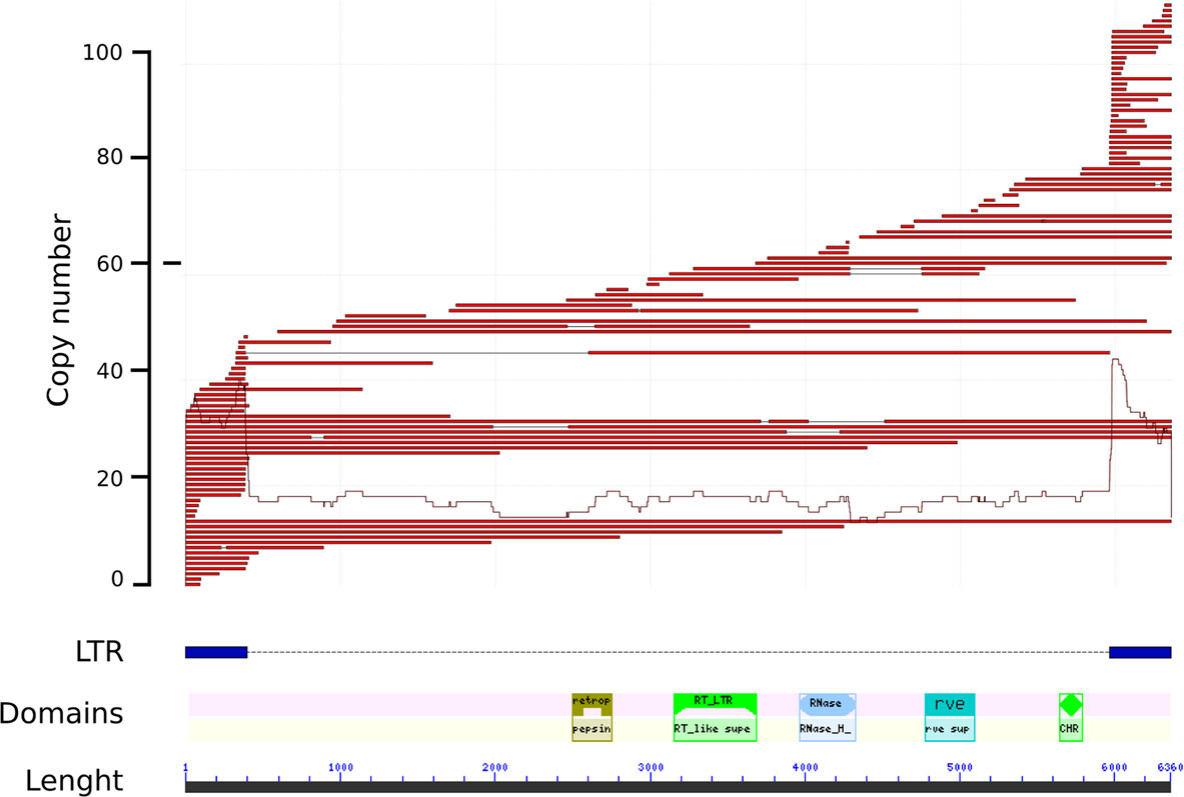
Abundance and structure of a *Chromoviridae* LTR-retrotransposon family of *C. olivacea.* The upper panel shows the mapping of the annotated genome copies of this family onto their consensus sequence. The lower panel shows a scheme of the structural and functional domains of this family: long terminal repeats (LTRs) are represented as blue rectangles; the internal domains shown are (from left to right): aspartate protease, reverse transcriptase, RNase, integrase, chromatin organization modifier

The TEs found belong to seven out of the nine TE orders described by Wicker* et al* [38]: LTR, DIRS (*Dictyostelium* Intermediate Repeat Sequences), PLE (Penelope-like Elements), LINE (Long Interspersed Nuclear Elements), SINE (Small Interspersed Nuclear Elements), TIR (Terminal Inverted Repeats) and Helitrons. Two of the orders (LTR and TIRS, which contain long terminal repeats or terminal inverted repeats, respectively) were present in the five species. Class I TEs were primarily responsible for the observed genome size differences—especially the elements belonging to LTR in the Gypsy superfamily, which accounted for more than 15% of the assembly in *S. lacrymans* and *P. tinctorius,* but less than 3% in *H. pinastri, C. olivacea* and *C. puteana.* Of all the LTR/Gypsy families detected by TEdenovo, we observed that those elements belonging to the *Chromoviridae* group (carrying a Chromatin organization domain, PF00385, in the N-terminal region after the integrase, Fig. 4) were the most abundant LTR-retrotransposons in these five species, ranging from 44 to 83% of the total Gypsy coverage. LTR-retrotransposons in the Copia superfamily were also particularly abundant in *S. lacrymans* and *P. tinctorius* (accounting for 2.4–6% of the total assembly size). Remarkably, non-coding LTR-retrotransposons such as TRIM (Terminal-repeat Retrotransposons In Miniature) and LARD (Large Retrotransposon Derivatives) were also found in three out of the five genomes, but in lower amounts (<1% of the genome, Table 4).

LINE, SINE, DIRS and PLE elements were also found in low copy numbers, but none of these were present in the five species. Regarding Class II transposons, TIR order was the most important in terms of abundance and copy number with elements encoding DDE transposases present in the five species. The second most important were MITEs (Miniature Inverted–repeat Transposable Elements) and other non-coding elements carrying structural features (classified as TIR/unknown in Table 1). Rolling-circle helitrons were found in *H. pinastri, S. lacrymans* and *P. tinctorius*, while putative Mavericks were present only in this latter one.

### Phylogenetic reconstruction of the LTR reverse-transcriptases

**Fig. 5:**
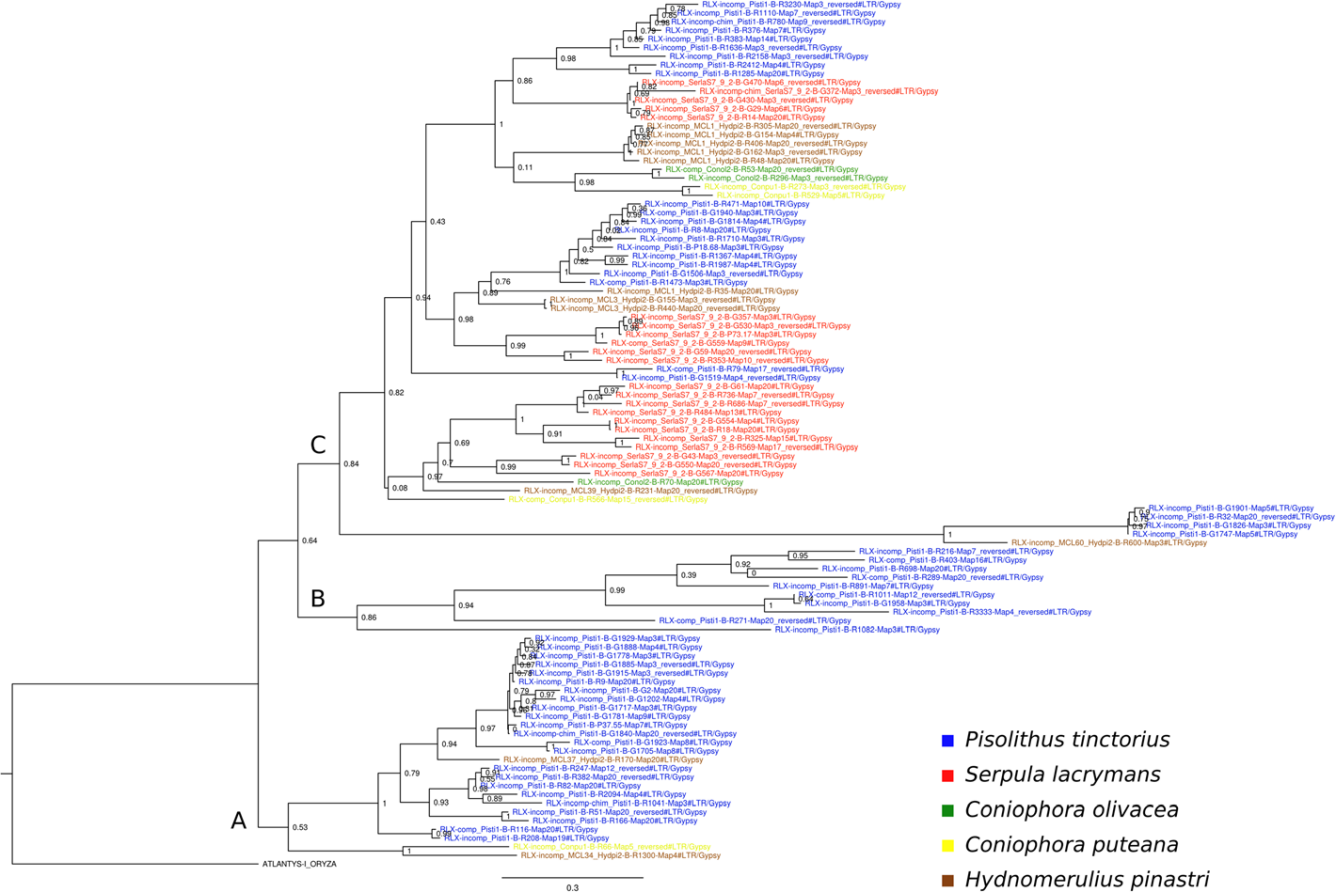
Maximum likelihood phylogeny of the Gypsy reverse-transcriptases found in the *C. olivacea*, *C. puteana*, *S. lacrymans*, *H. pinastri* and *P. tinctorius* (blue) genomes. SH (Shimodaira-Hasegawa) local support values are shown in branches. The reverse-transcriptase from *Oryza sativa* ATLANTIS-I family consensus (Repbase) was used as outgroup

To understand the phylogenetic relationship between the LTR-retrotransposon familes in the five analyzed genomes, we inferred a maximum likelihood phylogeny of the LTR reverse-transcriptases of the Gypsy consensus sequences (Fig. 5). Three main clades were obtained (A, B and C). Clades A and B were formed, almost exclusively, by families found in the *P. tinctorius* genome. Moreover, while clade B is formed mostly by distantly related families, the profile of clade A suggests that an important fraction of the families underwent recent diversification. All LTR families found in the other four species grouped in clade C along with the remaining families of *P. tinctorius*. This clade contained several retrotransposon sub-clades sharing closely related families from three to five species.

### Age of the LTR-retrotransposon amplification bursts in the Boletales

**Fig. 6:**
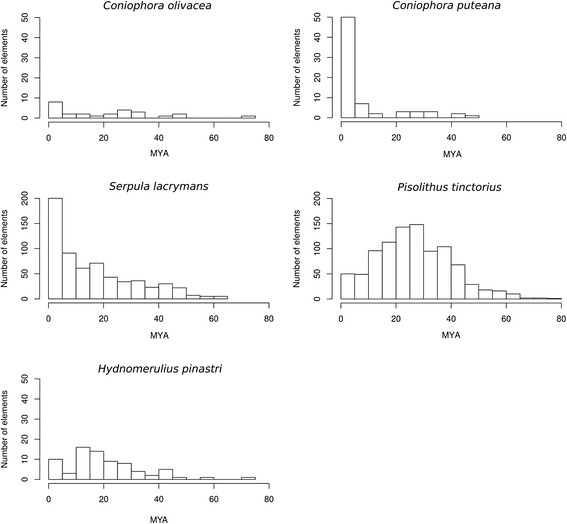
Estimated insertion age of the LTR-retrotransposons found in *C. olivacea, C. puteana, S. lacrymans, H. pinastri* and *P. tinctorius.* MYA = million years ago

LTR-retrotransposons carrying conserved domains as well as intact Long Terminal Repeats (putative autonomous elements) were subjected to further study to investigate their amplification dynamics over the course of evolution. Based on the nucleotide divergence between the two LTRs, we estimated the time of insertion of each element using a substitution rate of 1.05 × 10^−9^ nucleotide substitutions per site per year. The number of intact, putative autonomous LTR-retrotransposons varied greatly in the five species ranging from 26 elements in *C. olivacea* to 944 in *P. tinctorius.* The LTR profiles of *C. olivacea, C. puteana* and *S. lacrymans* showed recent peaks of amplification with insertion dates at 0–5 million years (MY). LTR amplification in *H. pinastri* showed a peak at 10–15 MY ago, whereas the profile of *P. tinctorium* pointed to a much older amplification burst showing a maximum peak at 25–30 MY ago and few recent retrotransposition events (Fig. 6).

## Discussion

### Genomic and proteomic characteristics of *C. olivacea*

We report the 39.07 Mb draft genome assembly and annotation of brown-rot basidiomycete *C. olivacea*. In terms of genome size, this species is slightly smaller than *C. puteana*, but it falls in the range of other brown-rot basidiomycetes such as *Hydnomerulius pinastri* (38.3 Mb) [4] or *Serpuyla lacrymans* (47.0 Mb). As expected for closely related species, *C. olivacea* and *C. puteana* show macrosynteny, although due to the short scaffold lengths it is impossible to establish comparisons at a chromosome scale. We found very good conservation of protein-coding genes, although *C. olivacea* has up to 1,352 orphan genes—most of these are supported by structure and RNA evidence (i.e., no homology to any other known gene). In this sense, the higher number of annotated genes in *C. olivacea* relative to *C. puteana* is probably related to the higher amount of assembled RNA contigs used to assist the annotation of the former (resulting from the higher RNAseq depth). The presence of about 10% of orphan genes is common in fungal genomes, and these genes often lack an *in silico* functional annotation as we found for *C. olivacea* [39, 40].

Wood-decaying species require a complex enzymatic machinery to degrade lignin and obtain nutrients. According to the CAZy enzymes identified in the genome, the *C. olivacea* proteome carries the main signatures of canonical brown-rot: (i) it completely lacks Class II peroxidases—enzymes primarily involved in lignin degradation [41], and (ii) it carries a reduced set of enzymes involved in degradation of crystalline cellulose. In fact, its profile is very similar to that of *C. puteana,* displaying only minor differences in several enzyme groups. As previously seen in other wood-degrading fungi, the *in silico* secretome of *C. olivacea* is enriched in functions related to lignocellulose degradation [42]. Our analysis showed that most intracellular and secreted proteins are members of multi-gene families of diverse size originating from gene duplications. The number of gene families that could not be functionally annotated by standard similarity-based methods was high, a phenomenon that is frequently observed in fungi.

To overcome this drawback, we used an alternative approach that combines similarity with structural information (Phyre-2). We then assigned a putative function to two multi-gene families conserved across the basidiomycete phylogeny but for which a putative function had not been previously proposed. Of special interest is the newly identified family of putative copper-dependent lytic polysaccharide monooxygenases (AA9, LPMO). The LPMOs are recently discovered enzymes used by microbes to digest crystalline polysaccharides [43]. They increase the saccharification yield of commercial enzyme cocktails [44]. Nevertheless, despite the promising results obtained *in silico*, experimental assays will be necessary to confirm the function of the members of this newly described gene family.

### Impact of TEs in the evolution of Boletales genomes

The results of TE annotation in the five Boletales showed how different patterns of LTR-retrotransposon amplifications have shaped the architecture of their genomes. The expansion of LTR/Gypsy retrotransposons belonging to *Chromoviridae* occurred mainly in the species with large genomes, whereas the smaller genomes have a small amount of these families (ie, three families in *C. olivacea* and *C. puteana)*. Chromoviruses are the most common LTR-retrotransposons in fungi [45], and the key to their success might be the presence of a chromo-integrase, which is thought to guide the integration of these elements into heterochromatic regions [46]. Heterochromatin is gene-poor, and it is silenced by epigenetic mechanisms such as DNA methylation and RNAi [47]. Thus, integration of these elements in such regions would allow them to skip purifying selection and increase their probability to persist in the genome. In fact, this could be the reason for the longer prevalence of *Gypsy* over *Copia* LTR-retrotransposons in most fungal species—the latter tend to integrate at random locations including euchromatic regions where transposon fixation is more difficult [48]. The LTR-retrotransposon amplification bursts of the Boletales indicate that elements from both *Coniophora* species are young and thus putatively active, and the profile of *S. lacrymans* also indicates a very strong activity of young copies with a progressive decrease in the amplification signals of older elements. Our findings suggest that the latter three species are currently in a period of genome expansion. Despite the different profile of *H. pinastri* and *P. tinctorius* we cannot rule out the same hypothesis, as both assemblies contain high gap content (7.7% and 13.3%, respectively). This fact usually leads to an underestimation in the amount of young retrotransposons [6], as they are difficult to assemble due to their repetitive nature and high sequence identity. In fact, we show that due to this reason the assembly-based TE quantification underestimated LTR content in *C. olivacea* in comparison to non-assembly based quantification (Additional file 2: Table S1)*.* The profile of *P. tinctorius* is intriguing. This ectomycorrhizal (ECM) species undergoes a massive expansion of LTR-retrotransposons in the Gypsy superfamily (similar to that found for other symbiotic species in Agaricomycotina [7, 49]; however, the majority of elements are very old (20–40 MY) and still carry structural and coding domains necessary for transposition. The phylogeny of Gypsy reverse-transcriptases suggests that many *P. tinctorius*-specific families are distantly related to the other four species. In fact, its impressive retrotransposon content might be partially explained by the amplification and diversification of ancestral families (giving rise to clades A and B in Fig. 5). Our phylogenetic reconstruction suggests that such ancestral families were also present in other boletales but didn’t proliferate in the genome (ie, *H. pinastri or C. puteana*). Whether genome defense mechanisms or lifestyle constraints are responsible of this phenomenon is still to be demonstrated. In this regards, it is interesting to note that the LTR-mediated genome amplification of *P. tinctorius* roughly coincides with the estimated origins of ECM symbiosis in Boletales [4]. Of the four Class I TE orders found, only the LTR elements were present in the five species. The most plausible scenario is that the elements from the other three orders (DIRS, LINE, and PLE) were lost by random drift in some of the species. Alternatively, they might be present in some genomes but in the form of very ancient and degenerated copies that are not detectable. Similarly, this patchy distribution was also found in class II elements (ie, helitrons were absent in the *Coniophora* genus and present in the remaining three species). Previous studies have shown that besides the conserved presence of LTR and TIR orders, the remaining TE groups tend to be present in variable amounts in basidiomycetes [6].

## Conclusions

In this study we present the draft genome sequence and annotation of the brown-rot fungi *Coniophora olivacea*, along with a comparative analysis with *C. puteana* and other members of the Boletales order. Our results show evidence of macrosynteny and conservation in the protein coding genes of the two species. The functional analysis of *C. olivacea* secretome showed that it displays the main signatures of a canonical brown-rot, and uncovered a new family of putative LPMOs widely conserved in basidiomycota. The annotation of transposable elements revealed a particular contraction in these two species in comparison to other Boletales, mainly due to the differential expansion of *Chromoviridae* LTR-retrotransposons. By analyzing the distribution of insertion ages and phylogenetic relationships of these elements we show that these LTR-retrotransposons have played a key role in the genome expansion experienced by certain species in the Boletales order.

## Additional files

Additional file 1: Text S1. Supplementary methods. (DOCX 16 kb)

Additional file 2: Table S1. Comparison of TE content estimation from REPET and Repeatexplorer. (XLSX 9 kb)

Additional file 3: Figure S1. Snapshot of synteny dot plot between *C. olivacea* and *C. puteana. (TIFF 582 kb)*

Additional file 4: Table S2. Comparison of CAZy proteins annotated in *C. olivacea* and *C. puteana. (XLSX 7 kb)*

Additional file 5: Table S3. Protein IDs of genes belonging to the 70 gene families with more than four members. (XLSX 9 kb)

Additional file 6: Dataset S1. TE annotation in *C. olivacea.* Classification information at the order level is included in the output format of PASTEC*. (GFF3 1342 kb)*

Additional file 7: Dataset S2. TE annotation in *C. puteana.* Classification information at the order level is included in the output format of PASTEC*. (GFF3 1273 kb)*

Additional file 8: Dataset S3. TE annotation in *H. pinastri.* Classification information at the order level is included in the output format of PASTEC*. (GFF3 1674 kb)*

Additional file 9: Dataset S4. TE annotation in *S. lacrymans.* Classification information at the order level is included in the output format of PASTEC*. (GFF3 8198 kb)*

Additional file 10: Dataset S5. TE annotation in *P. tinctorius.* Classification information at the order level is included in the output format of PASTEC*. (GFF3 12,724 kb)*

## Abbreviations

AA: Auxiliar activity
CAZYs: Carbohydrate-active enzymes
CBM: Carbohydrate-binding modules
CE: Carbohydrate esterases
CEGMA: Core Eukaryotic Genes Mapping Approach
DIRS: Dictyostelium intermediate repeat sequence
ECM: Ectomycorrhizal
GH: Glycoside hydrolase
GO: Gene Ontology
GPI: Glycosylphosphatidylinositol
HMM: Hidden Markov Models
Kb: Kilobase
KEGG: Kyoto Encyclopedia of Genes and Genomes
KOG: Eukaryotic Orthologous Groups
LARD: Large retrotransposon derivative
LINE: Long interspersed nuclear elements
LPMO: Lytic polysaccharide monooxygenases
LTR: Long Terminal Repeats
Mb: Megabase
MITE: Miniature inverted-repeat transposable elements
MY: Million years
PCWDE: Plant cell wall-degrading enzymes
PLE: Penelope-like elements
PSI: Position-Specific Iterated
RBH: Reciprocal best hit
RNAi: RNA interference
RV: Reverse-transcriptase
SH: Shimodaira-Hasegawa
SMY: Sucrose, malt, yeast
SRA: Sequence Read Archive
TEs: Transposable elements
TIR: Terminal inverted repeats
TRIM: Terminal-repeat retrotransposon in miniature
tRNA: transfer RNA
