# Supplementary material for: Comparative genomics of Coniophora olivacea reveals different patterns of genome expansion in Boletales
Source: BMC Genomics. 2017 Nov 16;18:883. doi: 10.1186/s12864-017-4243-z (PMC5689174; doi:10.1186/s12864-017-4243-z)
Supplement: Supplementary file 1 — Text S1. Supplementary methods. (DOCX 16 kb) [file 12864_2017_4243_MOESM1_ESM.docx]

**Supplementary methods**

**Nuclear assembly**

All raw Illumina sequence data was filtered for artifact/process contamination using the JGI QC pipeline: BBDuk (filterk=27, trimk=27; https://sourceforge.net/projects/bbmap/) was used to remove Illumina adapters, known Illumina artifacts, phiX and quality-trim both ends to Q12. Resulting reads containing more than one 'N', or with quality scores (before trimming) averaging less than 8 over the read, or length under 40bp after trimming, were discarded. Remaining reads were mapped to a masked version of human HG19 with BBMap, discarding all hits over 93% identity. CLIP PE, LFPE and CLRS are filtered with the linker sequences. An automated attempt was made to reassemble any potential organelle (mitochondrion) from the filtered reads and remove any organelle-matching reads with kmer matching against the resulting contigs with an in-house pipeline (organelle assembly section). An initial assembly of the target genome was generated using the resulting nonOrganelle reads using VelvetOptimiser version 2.1.7 (https://github.com/Victorian-Bioinformatics-Consortium/VelvetOptimiser.git) with Velvet version 1.2.07 [1] using the following parameters; "--s 61 --e 97 --i 4 --t 4, --o "-ins_length 250 -min_contig_lgth 500"". The resulting assembly was used to simulate 28X of a 2x100 bp 3000 +/- 300bp insert long mate-pair library with wgsim version 0.3.1-r13 (https://github.com/lh3/wgsim) using "-e 0 -1 100 -2 100 -r 0 -R 0 -X 0 -d 3000 -s 30". 25X of the simulated long mate-pair was then co-assembled together with 125X of the original Illumina filtered fastq with AllPathsLG release version R49403 [2] to produce the final nuclear assembly. Similar methodology, employing the UNITE rDNA database [3], was used to reassemble the ribosomal DNA from the filtered reads.

**Organelle assembly**

A 2 million read subsample was generated using BBtools version prod-v36.86 reformat.sh using "sampleseed=1". The subsampled data was additionally filtered for length and quality using an in-house tool fastqTrimmer (unpublished), with options "-b 5 -a 5 -l 101 -n 3 -p", and subsequently assembled together with Velvet version 2.1.7 [1] using velvetg "-cov_cutoff 20".  The resulting assembly was aligned to the NCBI refseq.mitochondrion database with BLAST megablast version 2.2.26 with a minimum percent identity of 80% to identify organelle. A secondary assembly was performed with Velvet version 2.1.7 using cov_cutoff, max_coverage, and exp_cov cutoffs defined from the coverages associated with the contigs previously identified as organelle. Read pairs providing linking support between the assembled contigs are identified by aligning the original input fastq to a version of the assembled contigs with all bases masked with N with exception to the terminal 300 bases, with bwa version 0.7.4-r385 [4] using "mem -t 16". The linking read pairs were used in conjunction with NCBI alignment results to refseq.mitochondrion to identify trusted organelle contigs. Main genome 18S ribosomal elements were identified by alignment to NCBI nt database with BLAST megablast version 2.2.26 with a minimum percent identity of 80% and excluded from the list. An enriched set of organelle reads was then created from the original input fastq reads by kmer matching with BBtools version prod-v36.86 BBDuk , using defaults, against the resulting white list of organelle contigs. Those that do not match the organelle contigs were output into a separate nonOrganelle fastq for downstream assembly. 125X of the enriched organelle matching read set was then coassembled together with 25X simulated 1000 +/- 50 bp insert long mate-pairs, generated from the organelle contigs with wgsim version 0.3.0 using "-d 1000 -s 50", with AllPathsLG release R46652 to produce a final mitochondrion assembly.

**Transcriptome sequencing and assembly**

Stranded cDNA libraries were generated using the Illumina Truseq Stranded RNA LT kit. mRNA was purified from 1µg of total RNA using magnetic beads containing poly-T oligos, fragmented and reversed transcribed using random hexamers and SSII (Invitrogen), followed by second strand synthesis. The fragmented cDNA was treated with end-pair, A-tailing, adapter ligation, and 10 cycles of PCR. The prepared libraries were quantified using KAPA Biosystem’s next-generation sequencing library qPCR kit and run on a Roche LightCycler 480 real-time PCR instrument. The quantified libraries were then multiplexed in equimolar proportions, and the pool of libraries was prepared for sequencing on the Illumina HiSeq-2500 platform. Using BBDuk, raw reads were evaluated for artifact sequence by kmer matching (kmer=25) allowing 1 mismatch, and detected artifact was trimmed from the 3' end of the reads. RNA spike-in reads, PhiX reads and reads containing any Ns were removed. Quality trimming was performed using the phred trimming method set at Q6. Finally, following trimming, reads under the length threshold were removed (minimum length 25 bases or 1/3 of the original read length - whichever is longer). Reads were assembled with Trinity [5] using the parameters “--grid_conf --jaccard_clip --seqType fq --normalize_reads --single --run_as_paired --min_per_id_same_path 95 --full_cleanup”.

**Estimation of TE content from Illumina Reads**

One million *C. olivacea* reads (coverage of 3.8X) were randomly sampled using Repeatexplorer sequence sampling tool [5]. The *de novo* TE library consisting of 51 consensus sequences (as obtained by TEdenovo, without TEannot iteration) was used as custom library. Repeatexplorer was run in the Galaxy server (www.repeatexplorer.org) with default parameters.

**References**

1. Zerbino DR, Birney E. Velvet: algorithms for de novo short read assembly using de Bruijn graphs. Genome Res. 2008; 18:821–9.

2. Gnerre S, MacCallum I, Przybylski D, Ribeiro FJ, Burton JN, Walker BJ, et al. High-quality draft assemblies of mammalian genomes from massively parallel sequence data. Proc. Natl. Acad. Sci. 2010;108:1513–8.

3. Kõljalg U, Nilsson RH, Abarenkov K, Tedersoo L, Taylor AFS, Bahram M, et al. Towards a unified paradigm for sequence-based identification of fungi. Mol. Ecol. 2013. 5271–7.

4. Li H, Durbin R. Fast and accurate short read alignment with Burrows-Wheeler transform. Bioinformatics. 2009; 25:1754–60.

5. Grabherr MG, Haas BJ, Yassour M, Levin JZ, Thompson DA, Amit I, Adiconis X, Fan L, Raychowdhury R, Zeng Q, et al. Full-length transcriptome assembly from RNA-Seq data without a reference genome. Nat Biotechnol. 2011; 29 (7): 644-652.

6. Novák P, Neumann P, Pech J, Steinhaisl J, Macas J. RepeatExplorer: a Galaxy-based web server for genome-wide characterization of eukaryotic repetitive elements from next-generation sequence reads. Bioinformatics. 2013;29:792–3
