# Supplementary figures and images for: Comparative genomics of Coniophora olivacea reveals different patterns of genome expansion in Boletales
Source: BMC Genomics. 2017 Nov 16;18:883. doi: 10.1186/s12864-017-4243-z (PMC5689174; doi:10.1186/s12864-017-4243-z)

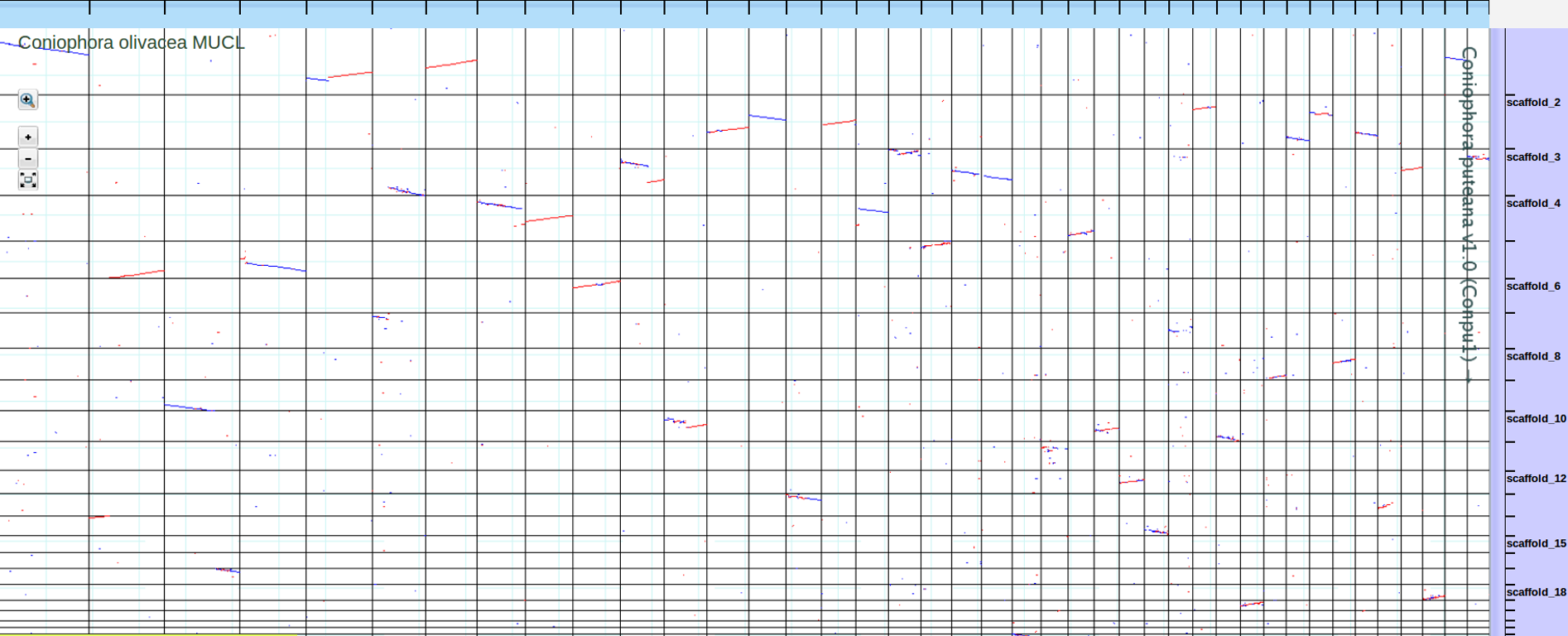

Supplement: Supplementary file 3 — Snapshot of synteny dot plot between C. olivacea and C. puteana. (TIFF 582 kb) [file 12864_2017_4243_MOESM3_ESM.tif]
